# Crystal structure and characterization of pyrroloquinoline quinone disodium trihydrate

**DOI:** 10.1186/1752-153X-6-57

**Published:** 2012-06-19

**Authors:** Kazuto Ikemoto, Hitoshi Sakamoto, Masahiko Nakano

**Affiliations:** 1 Niigata Research Laboratory, Mitsubishi Gas Chemical Company, Inc, 182 Tayuuhama Shinwari, Kita-ku, Niigata, Japan

## Abstract

**Background:**

Pyrroloquinoline quinone (PQQ), a tricarboxylic acid, has attracted attention as a growth factor, and its application to supplements and cosmetics is underway. The product used for these purposes is a water-soluble salt of PQQ disodium. Although in the past, PQQ disodiumpentahydrates with a high water concentration were used, currently, low hydration crystals of PQQ disodiumpentahydrates are preferred.

**Results:**

We prepared a crystal of PQQ disodium trihydrate in a solution of ethanol and water, studied its structure, and analyzed its properties. In the prepared crystal, the sodium atom interacted with the oxygen atom of two carboxylic acids as well as two quinones of the PQQ disodium trihydrate. In addition, the hydration water of the prepared crystal was less than that of the conventional PQQ disodium crystal. From the results of this study, it was found that the color and the near-infrared (NIR) spectrum of the prepared crystal changed depending on the water content in the dried samples.

**Conclusions:**

The water content in the dried samples was restored to that in the trihydrate crystal by placing the samples in a humid environment. In addition, the results of X-ray diffraction (XRD) and X-ray diffraction-differential calorimetry (XRD-DSC) analyses show that the phase of the trihydrate crystal changed when the crystallization water was eliminated. The dried crystal has two crystalline forms that are restored to the original trihydrate crystals in 20% relative humidity (RH). This crystalline (PQQ disodium trihydrate) is stable under normal environment.

## Introduction

**Figure 1:**
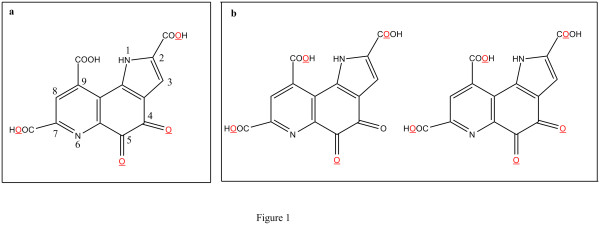
**Chemical structure of PQQ. Sodium interacts with the oxygen present at the underlined position.** (**a**) PQQ disodium trihydrate. 2- and 7-COOH groups take anion forms (2- and 7-COO-). (**b**) PQQ disodium pentahydrate. Two crystallographically independent conformation PQQ.

Pyrroloquinoline quinone (PQQ; 4,5-dihydro-4,5-dioxo-1 H-pyrrolo[2,3-f]quinoline- 2,7,9-tricarboxylic acid) (Figure 1a) is a water-soluble quinone that was first identified as a non-covalently prosthetic group in some bacterial glucose- or alcohol dehydrogenases [1,2]. It is interesting to note that trace amounts of PQQ have been found not only in microorganisms but also in humans and in the organs and tissues of rats, with the highest amount being found in human milk [3,4]. In addition, trace amounts of PQQ have also been found in daily foods and beverages [5,6]. In recent years, PQQ has been receiving considerable attention owing to its several interesting physiological functions [7,8]. PQQ stimulates mitochondrial biogenesis through cAMP response element-binding protein phosphorylation and an increase in PGC-1α expression [9,10]. In addition, PQQ promotes the regeneration of peripheral and central nerves. From the results of the *in vitro* experiment, it was found that PQQ enhances the nerve growth factor (NGF)—a neurotrophic factor responsible for the maintenance and development of peripheral nerves [11]. Further, the regeneration of a transected sciatic nerve in an *in vivo* rat model was demonstrated [12]. A recent study suggests that PQQ protects against secondary damage by attenuating the inducible nitric oxide synthase (iNOS) expression following a primary physiological injury to the spinal cord [13].

Previous studies showed that PQQ and reduced PQQ are antioxidative in nature [14,15]. Their antioxidative function was observed in culture cells and rats [16,17]. PQQ disodium of an ionic material is often used as a water-soluble salt because the tricarboxylic acid type has low solubility. In many cases, organic molecules exist in a crystalline state in several different forms such as polymorphs or hydrates. The crystalline form of an organic molecule is important from the viewpoint of its application in food and pharmaceutical products. However, the polymorphs of PQQ disodium crystalline have not been studied. Thus far, the crystal structures of an acetone adduct [2], a metal complex [18], an ester [19], and PQQ disodium pentahydrate [20] have been reported.

A single crystal of PQQ disodium pentahydrate was obtained by the slow evaporation of phosphate buffer at 20°C. However, the crystal of PQQ disodium pentahydrate obtained by this method is unstable because it contains a large amount of water. Therefore, owing to this drawback, the abovementioned method is not suitable for the industrial manufacture of crystals of PQQ disodium pentahydrate. On the other hand, a powder of PQQ disodium was industrially manufactured by adding ethanol (poor solvent) to a water solution of PQQ disodium [21]. However, this product, which included ethanol, had low crystallinity.

We successfully prepared PQQ disodium trihydrate with low water content. The structure of the prepared crystal was analyzed by single crystal X-ray diffraction.

Further, we studied the effect of the water content on the crystalline form, color, and near-infrared (NIR) spectrum of the prepared crystal.

## Results and discussion

### Single crystal analysis

**Figure 2:**
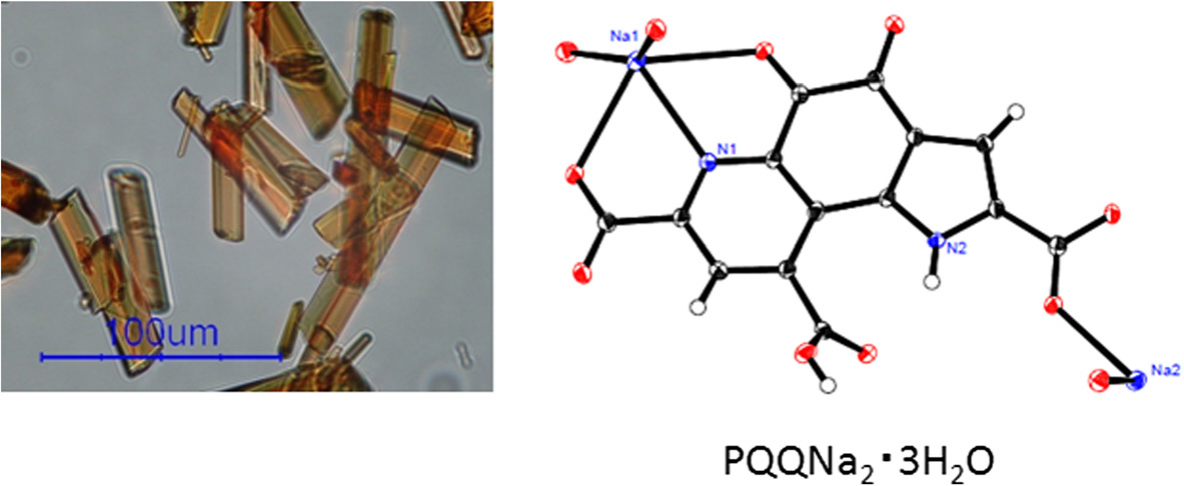
**Left: PQQ disodium trihydrate crystal observed under a microscope.** Right: Molecular structure of PQQ disodium trihydrate (ORTEP).

A molecule of PQQ was separated from the culture of a microbe, and PQQ trisodium was salted out at neutral pH. The pH of the suspension of PQQ trisodium in ethanol/water was reduced, and PQQ disodium was crystallized. The board-shaped crystal of PQQ disodium (Figure 2) exhibits high crystallinity; the result of single crystal X-ray diffraction analysis is shown in Figure 2, and Table 1 summarizes the supporting information. The obtained crystal is PQQ disodium trihydrate (type I), and it does not include ethanol. With regard to the crystals prepared by the conventional ethanol precipitation method, it is found that ethanol is present in the sediment and that the crystals exhibit poor crystallinity. As shown in Figure 2, Na^+^-1 forms ionic bonds with the oxygen atoms of the 7-COO- and 5-CO- groups, and Na^+^-2 forms an ionic bond with an oxygen atom of the 2-COO- group in PQQ. In addition, Na^+^-2 also forms a bond with the oxygen atom of 4-CO of the neighboring molecule. In PQQ disodium trihydrate, the 2- and 7-COOH groups take anion forms (2- and 7-COO-). Moreover, such bonding is not observed in 9-carboxylic acid (Figure 1a). A crystal of PQQ disodium pentahydrate comprises two types of molecules of PQQ (Figure 1b); and thus far, it has been observed that only one of those quinones did not form sodium and an ionic bond. The difference in the type of molecules leads to a difference in the lattice multiplier. Furthermore, the difference resulted in the production of our crystal at 3.195 Å with 3.607 Å, as well as resulted in the formation of pentahydrates in the interval between the shortest π planes.

**Table 1:** Crystal data for PQQdisodium trihydrate

Empirical Formula	C_14_H_10_N_2_Na_2_O_11_
Formula Weight	428.22
Crystal Color	Habit red, block
Crystal Dimensions	0.100 × 0.100 × 0.100 mm
Crystal System	triclinic
Lattice Parameters	Primitive, a = 7.6087(2) Å, b = 10.0963(2) Å, c = 11.4337(2) Å, α = 72.858(5) °, β = 88.015(7) °, γ = 82.627(6) °, V = 832.37(4) Å3
Space Group	P-1 (#2)
Z value	2
Dcalc.	1.708 g/cm^3^, F000 436.00

The crystal obtained was nothing but the PQQ disodium crystal, but with a low amount of water. This low hydration is effective in suppressing the growth of microbe and increasing the stability of the material when it is used in food and drugs.

### Drying and moisture absorption

**Figure 3:**
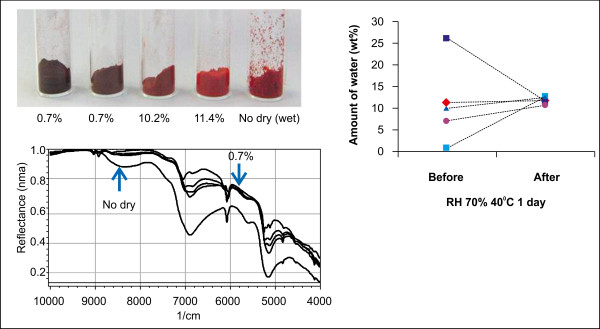
**Samples with different water content prepared by drying PQQ disodium trihydrate.** Images of the samples with different water content. Near-infrared (NIR) spectra of dried samples. All the samples with different water content show a water content of around 12% after being subjected to a relative humidity (RH) of 75% at 40°C for 1 day.

The PQQ disodium trihydrate crystal contains 12.7% water. The water included in the crystal can be eliminated by vacuum or heat drying. The crystal prepared in this study had a water content of 26.5%, and water was even found on the surface of the crystal. However, after this crystal was subjected to vacuum drying, its water content decreased drastically to only 0.7%. It was found that the color of the crystal changed considerably from red to dark brown with a decrease in the water content (Figure 3). The obtained crystal was subjected to colorimetric analysis; this analysis did not have an effect on the color of the crystal and it reduced the chroma. The results of this analysis showed that the change in the color of the crystal was linear.

Recently, near-infrared (NIR) analysis has been used for the quality control of food and drugs. An important point to note here is that the results of this method of analysis are also strongly influenced by the water content in a crystal. From the NIR spectrum of a crystal, it was found that a broad peak became sharp with a decrease in the water content (see Figure 3). The water content of the samples converged to approximately 12% when the samples were subjected to a humidity of 75% RH at 40°C for 1 day. After moisture absorption, the color and the NIR spectra of all the samples were identical to those of the original trihydrate crystal. It is interesting to note that a sample whose water content was 26.5% higher than that of the trihydrate crystal showed a water content of only 12%. This result indicated that the water outside the crystal evaporated and that the crystalline water remained. It is easy to change the weight of a very dry sample in a normal environment because the dried sample now becomes hygroscopic in nature.

### Water content and powder X-rays analysis

Techniques for carrying out structure determination directly from powder X-ray diffraction (XRD) data clearly provide the structural properties of a polycrystalline product in solid-state transformation. We studied the structure of the crystal of PQQ disodium trihydrate (type I) by XRD when it was subjected to the drying process. The temperature was continuously changed to examine a decrease in the water content and a change in the crystal structure.

**Figure 4:**
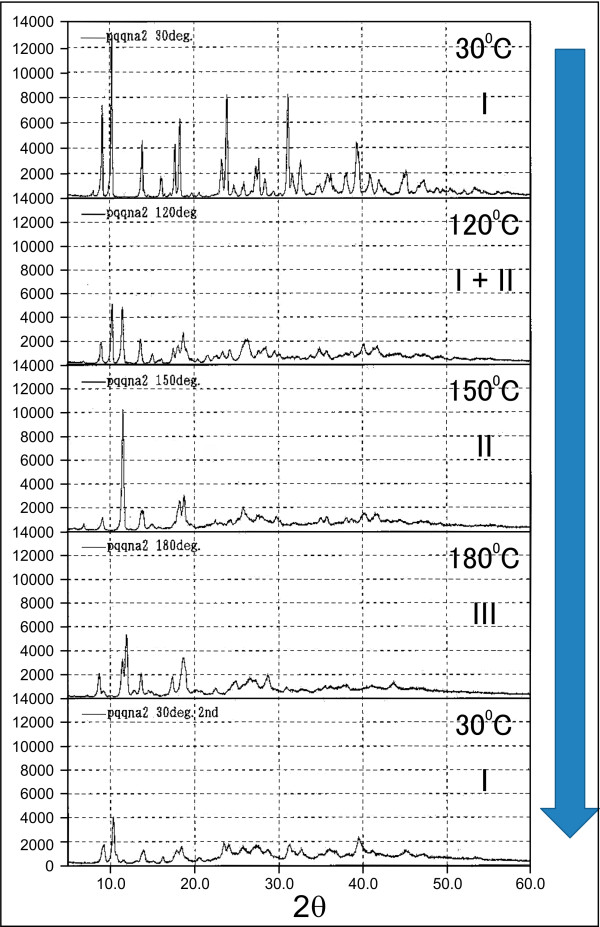
**Powder X-ray diffractometry (XRD) of PQQ disodium trihydrate in air.** The type I trihydrate crystal changed to the type II trihydrate crystal at150°C, which in turn changed to the type III crystal at 180°C. The crystal formed at 120°C is a mixture of type I and type II crystals. 2θ = type I: 9.1, 10.3, 13.8, 17.7, 18.3, 24.0, 27.4, type II: 11.4, 13.5, 18.0, 18.7, 26.0, 28.5, type III: 7.0, 11.5, 12.0, 17.4, 18.7.

The result of the effect of the variation in temperature on the water content is shown in Figure 4. The water included in the crystal can be eliminated by a general drying method. The crystal was subjected to crystallographic analysis during which the water content in the crystal changed with drying. In addition, the temperature was changed in the air to examine a decrease in the water content and a change in the crystal structure. In the XRD spectrum of the trihydrate crystal (type I), new peaks were observed at 120°C and the crystal also exhibited a new crystalline form. The type I crystal disappeared at 150°C, and a new crystal (type II) was formed. The type II crystal is capable of exhibiting higher symmetry than the type I crystal because its XRD spectrum shows few peaks. Furthermore, at temperatures higher than 180°C, a type III crystal was formed. It was observed that a peak of the type I crystal appeared when the temperature decreased to 30°C for 1 h under atmospheric conditions.

**Figure 5:**
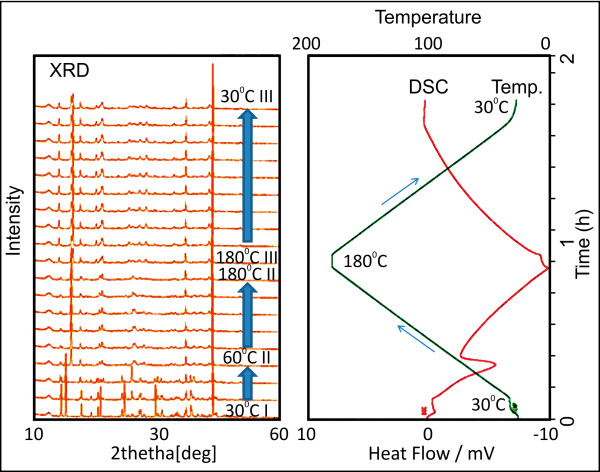
**XRD-DSC (left, XRD; right, DSC, temperature).** Temperature was changed from 30°C to 180°C and back to 30°C (3°C/min), and the second phase conversion occurred at 180°C.

In this study, the samples with different water content were measured by XRD. The samples with a water content of 10.2–26.5% were type I crystals. The samples with a water content of 7.0% were a mixture of type I and type II crystals, such as the sample obtained at 120°C. The samples with a water content of 0.7% were only type II crystals. After treatment in a humid environment, all the samples became type I crystals and contained around 12% water. As in the case of the color of the crystal, its structure was restored by the water content. The phase of the crystal undergoes conversion. Furthermore, simultaneous X-ray diffraction-differential scanning calorimetry (XRD-DSC) was performed for detailed analysis of the crystal phase. The temperature-dependent measurement of PQQ disodium trihydrate in nitrogen (no humidity) is shown in Figure 5. The graph shown on the right depicts the DSC result of a measurement (temperature, DSC curve), and the graph shown on the left depicts the occasional XRD profile. Temperature was changed from 30°C to 180°C and back to 30°C (3°C/min) in nitrogen. The XRD spectrum with heat absorption on the DSC curve at 40–85°C in the temperature-increasing process was changed, and the crystalline water was eliminated.

Amount of heat can be calculated from DSC curve. The analysis is shown below.

In heat absorption, 187 J/g, the peak top temperature was 73°C, and crystal water was eliminated. A slight change of DSC was measured at 180°C, and the conversion in the phase occurred at 180°C. In the subsequent low-temperature process, the absorption of the heat and a change in the XRD profile were not observed. The peak obtained after the DSC of the dehydration of the crystalline water was in good agreement with that of the type II crystal obtained from the type I crystal of trihydrate. Furthermore, the type II crystal was changed to type III crystal at 180°C. The mixture of type I and type II crystals could be observed in air, but not in nitrogen. The crystal structure did not change under dry condition. When the temperature of the type III crystal was lowered to room temperature under nitrogen atmosphere, the structure was maintained. However, the type III crystal structure changed to type I in the air.

The type II crystal is an anhydride; the experiment performed in this study shows that the phase of the type II crystal changed at 180°C, at which point a new type III crystal was formed, with a structure that differed from that of the type II crystal. The hydration of the type III crystal at 35°C was measured by XRD-DSC in a moisture absorption process.

**Figure 6:**
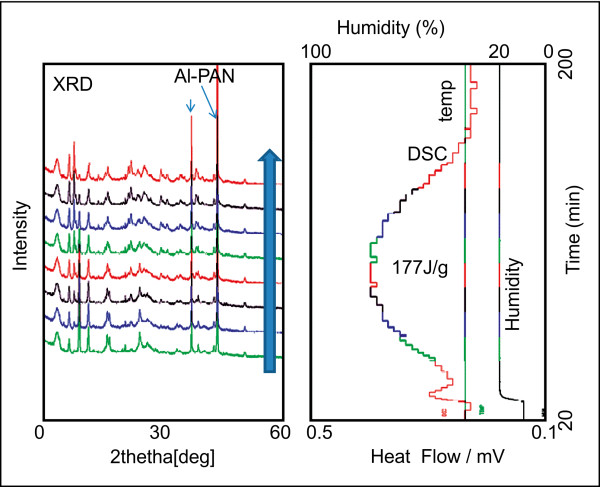
**XRD-DSC (left; XRD; right, DSC, temperature). The X-ray Diffraction changed following the water absorption at 10% humidity.** Crystal Type III changed to trihydrate crystal type I.

The humidity was increased from 0 to 90% RH, and the sample was measured. The result of this measurement is shown in Figure 6. A peak in the DSC curve of water absorption was observed when the humidity was increased to 20% RH. This peak lasted for approximately 130 min, and the crystallization was observed when the XRD profile gradually changed. When the humidity was increased to 30% RH, change in DSC and in the XRD profile were not observed. It was inferred that the type III crystal transformed to the type I trihydrate crystal. The calorific value of water absorption was 177 J/g, which shows the type I of trihydrate crystal is 75.8 kJ/M more stable than type III in normal air.

**Figure 7:**
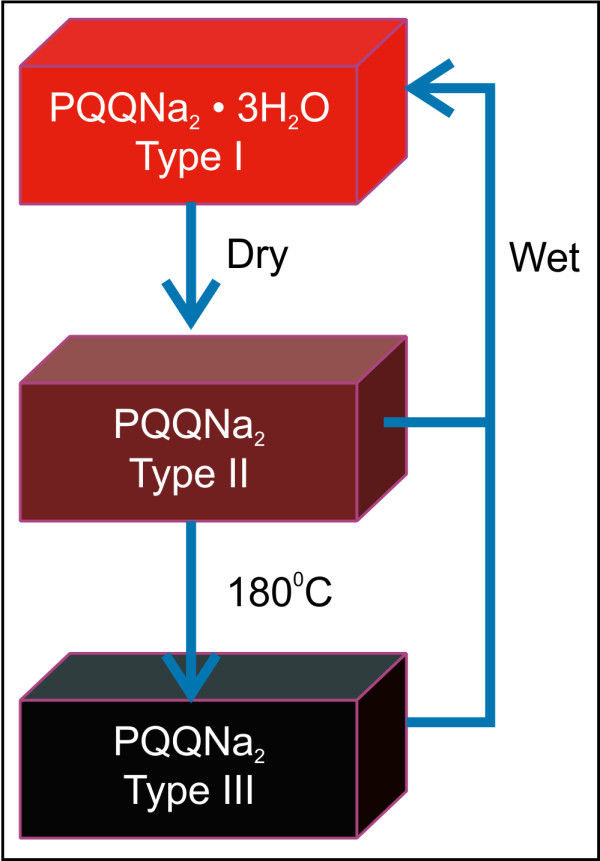
**Trihydrate crystal type I changed to type II under vacuum, and further changed to type III at 180°C.** Crystal type II and III changed to type I under humidity.

First, we thought that the change in the crystalline form advanced from trihydrates to mono- or di-hydrates in the type II crystal. It was thought that due to high absorbency, type III crystal was not observed in normal dry air. However, phase transition occurs in the type II crystal after a dehydration process on XRD-DSC in nitrogen. Moreover, the type II crystal was transformed to a type III crystal at a high temperature of 180°C. In other words, the type II crystal is an anhydride crystal, and the type III crystal is an anhydrous crystal. A mixture of a type I and type II crystal can be obtained in air with humidity. Details on non-aqueous crystal structures are not yet understood.

We expected that on elimination of the water, the crystals would become either amorphous or porous. However, contrary to our expectation, the crystals were not amorphous. The surface area of these crystals was measured by nitrogen absorption. From the results of this measurement, it was found that the surface area of all the crystals was less than the BET surface area of 2 m^2^/g. This result shows that the crystal structures are not porous and that they change considerably with the removal of water.

We also found that the water intercalated in to a crystal to result in the formation of trihydrate. The following conclusions can be drawn from the abovementioned results (Figure 7). The color, the NIR spectrum attributed to drying, and the structure of the type II trihydrate crystals change.

Furthermore, phase transition of the type II crystal occurs at 180°C—a temperature at which the type III crystal is formed. The crystal can either be of type I, a mixture of type I and type II, and type II, depending on the humidity in air. The type III crystal can also be obtained at room temperature by reducing the phase transition temperature of the type II crystal in the dry state. The trihydrates were found to be stable in a humid environment, whereas the type II and type III crystals were unstable in this environment in that they both returned to their original phase. Trihydrate crystals can easily absorb as much as 20% water from a humid environment, contributing to their stability under humidity. Therefore, a trihydrate crystal exhibits suitable properties such that it can be used as a commercial product.

## Summary

A single crystal of PQQ disodium trihydrate was prepared. Two carboxylic acid and two quinone form ionic bonds with oxygen and sodium in this crystal. The water content of this crystal had a significant effect on the color and the change in the NIR absorption spectrum; the prepared crystal was found to undergo phase transition at different temperatures. This crystal exhibits a crystalline form that 180° or more different from that of an anhydride. The dried sample becomes a trihydrate crystal at 20% humidity, with the color and NIR spectrum same as trihydrate crystals. In case of this crystal, trihydrates are stable and their absorbency is low. For their analysis and stability of their color, extensive drying should be avoided. This crystalline (PQQ disodium trihydrate) is stable under normal environment.

## Experimental methods

### Preparation of PQQ disodium crystals

The PQQ was prepared by fermentation and was purified by column chromatography. The PQQ trisodium solid was precipitated by adding NaCl at pH 7.5. A suspension of this solid was obtained with the addition of a 50% by volume ethanol-water solution. Then, hydrochloric acid was slowly added to this suspension, following which the suspension was crystallized at pH 3.5, and a PQQ disodium crystal was obtained. The samples with different content of crystallization water were prepared under different vacuum conditions.

### Water content analysis

The sample was heated to 180°C, and the water that was eliminated from the crystal was analyzed by the Karl Fischer method using a Metrohm 831 KF coulometer.

### Single crystal X-ray structure analysis

Rigaku R-AXIS RAPID, radiation Cu-Kα voltage/current: 40 kV/30 mA, 23.0°C. Crystallography data have been deposited additional files, Additional file 1: Check cif report, Additional file 2: Original X-ray analysis data by CIF format.

Powder X-ray diffractometry (XRD)

M18XCE, manufactured by MAC Science, Co. Ltd.

Cu-Kα 40 kV/100 mA, divergence slit: 1°, scattering slit: 1°, receiving slit: 0.3 mm, scan speed: 4.000°/min, sampling width: 0.02°.

XRD-DSC

Rigaku SmartLab (sample horizontal model multi-purpose X-ray diffraction)

High-speed one-dimensional detector D/teX Ultra

X-ray source Cu-Kα (40 kV/50 mA) with a scanning speed of 5°/min

0% RH, 30–180°C (3°C/min) Step 2: 35°C, 0–90% RH atmosphere dry-wet N_2_.

## Competing interests

The authors declare that they have no competing interests.

## Authors’ contribution

KI has coordinated the study, carried out the experimental work and drafted the manuscript. HS contributed the synthesis of powder of different water content. MN and KI discussed and analyzed the results. All authors have read and approved the final manuscript.

## Supplementary Material

Additional file 1 Check CIF/PLATON report. Click here for file

Additional file 2 Original X-ray analysis data. Click here for file
