# Supplementary material for: Crystal structure and characterization of pyrroloquinoline quinone disodium trihydrate
Source: Chem Cent J. 2012 Jun 19;6:57. doi: 10.1186/1752-153X-6-57 (PMC3541126; doi:10.1186/1752-153X-6-57)

checkCIF/PLATON report

Structure factors have been supplied for datablock(s) sc0909b_a_proc

No syntax errors found. CIF dictionary Interpreting this report

Datablock: sc0909b_a_proc

Bond precision: C-C = 0.0048 A Wavelength=1.54187

Cell:	a=7.60872(14)	b=10.09635(18)	c=11.4337(2)
	alpha=72.858(5)	beta=88.015(7)	gamma=82.627(6)
Temperature:	296 K		

	Calculated	Reported
Volume	832.38(4)	832.37(4)
Space group	P -1	P -1
Hall group	-P 1	-P 1
Moiety formula	C14 H4 N2 Na2 O11	C14 H10 N2 Na2 O11
Sum formula	C14 H4 N2 Na2 O11	C14 H10 N2 Na2 O11
Mr	422.17	428.22
Dx,g cm-3	1.684	1.708
Z	2	2
Mu (mm-1)	1.737	1.740
F000	424.0	436.0
F000’	426.17	
h,k,lmax	9,12,13	9,12,13
Nref	3055	2994
Tmin,Tmax	0.840,0.840	0.679,0.840
Tmin’	0.840	

Correction method= MULTI-SCAN

Data completeness= 0.980 Theta(max)= 68.240

R(reflections)= 0.0475(2545) wR2(reflections)= 0.1429(2994)

S = 1.085 Npar= 262

The following ALERTS were generated. Each ALERT has the format
test-name_ALERT_alert-type_alert-level.
Click on the hyperlinks for more details of the test.

Alert level A

PLAT430_ALERT_2_A Short Inter D...A Contact O3 .. O3 .. 2.47 Ang.
PLAT902_ALERT_1_A No (Interpretable) Reflections found in FCF .... !

Alert level B

```
PLAT430_ALERT_2_B Short Inter D...A Contact  O4     ..  O11     ..       2.79 Ang.
PLAT430_ALERT_2_B Short Inter D...A Contact  O4     ..  O10     ..       2.83 Ang.
```

Alert level C

```
CHEMW03_ALERT_2_C The ratio of given/expected molecular weight as
            calculated from the _atom_site* data lies outside
            the range 0.99 <> 1.01
           From the CIF: _cell_formula_units_Z                    2
           From the CIF: _chemical_formula_weight           428.22
           TEST: Calculate formula weight from _atom_site_*
           atom     mass    num     sum
           C        12.01   14.00  168.15
           H         1.01    4.00    4.03
           O        16.00   11.00  175.99
           N        14.01    2.00   28.01
           Na       22.99    2.00   45.98
           Calculated formula weight             422.17
PLAT041_ALERT_1_C Calc. and Reported SumFormula    Strings  Differ          ?
PLAT042_ALERT_1_C Calc. and Reported MoietyFormula Strings  Differ          ?
PLAT043_ALERT_1_C Check Reported Molecular Weight ................     428.22
PLAT068_ALERT_1_C Reported F000 Differs from Calcd (or Missing)...          ?
PLAT340_ALERT_3_C Low Bond Precision on  C-C Bonds ...............     0.0048 Ang
PLAT369_ALERT_2_C Long   C(sp2)-C(sp2) Bond  C9     -   C10    ...       1.55 Ang.
PLAT482_ALERT_4_C Small D-H..A Angle Rep for N2     ..  O7      ..      95.50 Deg.
```

Alert level G

```
FORMU01_ALERT_2_G  There is a discrepancy between the atom counts in the
            _chemical_formula_sum and the formula from the _atom_site* data.
            Atom count from _chemical_formula_sum:C14 H10 N2 Na2 O11
            Atom count from the _atom_site data:  C14 H4 N2 Na2 O11
CELLZ01_ALERT_1_G Difference between formula and atom_site contents detected.
CELLZ01_ALERT_1_G WARNING: H atoms missing from atom site list. Is this intentional?
           From the CIF: _cell_formula_units_Z    2
           From the CIF: _chemical_formula_sum  C14 H10 N2 Na2 O11
           TEST: Compare cell contents of formula and atom_site data

           atom    Z*formula  cif sites diff
           C         28.00     28.00    0.00
           H         20.00      8.00   12.00
           N          4.00      4.00    0.00
           Na         4.00      4.00    0.00
           O         22.00     22.00    0.00
CHEMS02_ALERT_1_G  Please check that you have entered the correct
            _publ_requested_category classification of your compound;
            FI or CI or EI for inorganic; FM or CM or EM for metal-organic;
            FO or CO or EO for organic.
            From the CIF: _publ_requested_category   CHOOSE FI FM FO CI CM CO or
            From the CIF: _chemical_formula_sum:C14 H10 N2 Na2 O11
PLAT004_ALERT_5_G Info: Polymeric Structure Found with Dimension .          3
PLAT005_ALERT_5_G No _iucr_refine_instructions_details in CIF ....          ?
PLAT007_ALERT_5_G Note: Number of Unrefined D-H Atoms ............          2
PLAT335_ALERT_2_G Check Large C6 Ring    C-C Range C4     -C11           0.16 Ang.
PLAT808_ALERT_5_G No Parseable SHELXL Style Weighting Scheme Found          !
PLAT860_ALERT_3_G Note: Number of Least-Squares Restraints .......        245
```

---

2 **ALERT level A** = Most likely a serious problem - resolve or explain
2 **ALERT level B** = A potentially serious problem, consider carefully

```
 8 ALERT level C = Check. Ensure it is not caused by an omission or oversight
10 ALERT level G = General information/check it is not something unexpected

 8 ALERT type 1 CIF construction/syntax error, inconsistent or missing data
 7 ALERT type 2 Indicator that the structure model may be wrong or deficient
 2 ALERT type 3 Indicator that the structure quality may be low
 1 ALERT type 4 Improvement, methodology, query or suggestion
 4 ALERT type 5 Informative message, check
```

It is advisable to attempt to resolve as many as possible of the alerts in all categories. Often the minor alerts point to easily fixed oversights, errors and omissions in your CIF or refinement strategy, so attention to these fine details can be worthwhile. In order to resolve some of the more serious problems it may be necessary to carry out additional measurements or structure refinements. However, the purpose of your study may justify the reported deviations and the more serious of these should normally be commented upon in the discussion or experimental section of a paper or in the "special_details" fields of the CIF. checkCIF was carefully designed to identify outliers and unusual parameters, but every test has its limitations and alerts that are not important in a particular case may appear. Conversely, the absence of alerts does not guarantee there are no aspects of the results needing attention. It is up to the individual to critically assess their own results and, if necessary, seek expert advice.

Publication of your CIF in IUCr journals

A basic structural check has been run on your CIF. These basic checks will be run on all CIFs submitted for publication in IUCr journals (*Acta Crystallographica*, *Journal of Applied Crystallography*, *Journal of Synchrotron Radiation*); however, if you intend to submit to *Acta Crystallographica Section C* or *E*, you should make sure that full publication checks are run on the final version of your CIF prior to submission.

Publication of your CIF in other journals

Please refer to the *Notes for Authors* of the relevant journal for any special instructions relating to CIF submission.

PLATON version of 15/02/2012; check.def file version of 10/02/2012

Datablock sc0909b_a_proc - ellipsoid plot

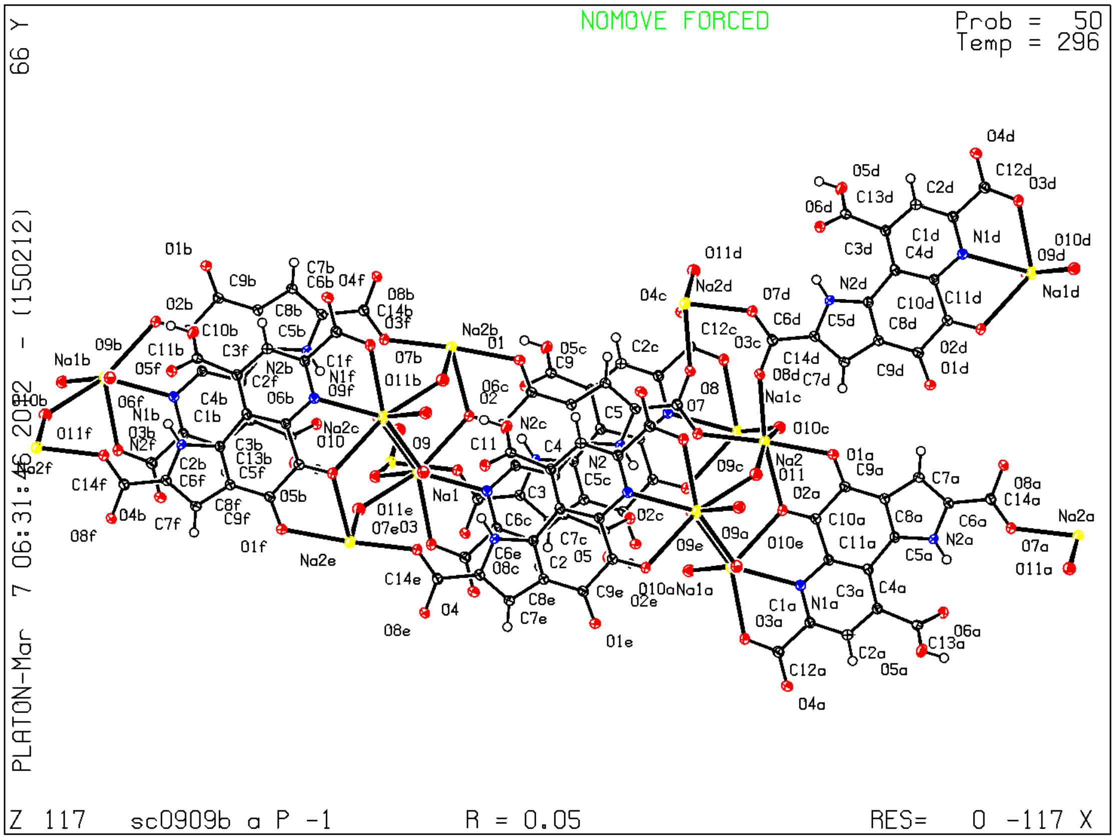

Supplement: Additional file 1 — Check CIF/PLATON report. [file 1752-153X-6-57-S1.pdf]
